# Do Glazed Ceramic Pots in a Mexico-US Border City Still Contain Lead?

**DOI:** 10.1155/2014/474176

**Published:** 2014-10-29

**Authors:** Ana M. Valles-Medina, Angel I. Osuna-Leal, Maria Elena Martinez-Cervantes, Maria Carmen Castillo-Fregoso, Martha Vazquez-Erlbeck, Alfonso Rodriguez-Lainz

**Affiliations:** ^1^School of Medicine and Psychology, Autonomous University of Baja California, 14418 Calzada Tecnologico, 22390 Tijuana, BCN, Mexico; ^2^MSN/FNP Program, United States University, 830 Bay Boulevard, Chula Vista, CA 91911, USA; ^3^School of Public Health, San Diego State University, 5500 Campanile Drive, San Diego, CA 92182, USA

## Abstract

In order to identify the presence of lead in glazed ceramic pots in a Mexico-US border city, 41 clay pots were sampled. The pots were purchased in several establishments located in different geographical areas of the city. The presence of lead was determined using LeadCheck Swabs. Most (58.5%) of the pots were from the State of Jalisco and 24.4% were of unknown origin. Only 4 pots did not contain varnish and were lead-negative. Thirty-seven (81.1%) of the glazed pots were lead positive. Among the lead-negative pots, 4 showed the label “this pot is lead-free.” Thus, if we consider the remaining 33 glazed pots without the “Lead-Free” label, 90.9% were lead-positive and only 9.1% were lead-negative. We also found that earthenware glazed utensils without the “Lead-Free” label were 1.6 times more likely to contain lead (OR: 1.6, 95% CI 1.0–2.5), *P* = 0.003. We concluded that lead was detected in almost all acquired food containers. Government interventions in Mexico have focused on training manufacturers to make lead-free glazed ceramics but it has been difficult to eradicate this practice. Educational interventions to make and acquire lead-free glazed ceramics should be targeted to both sellers and buyers.

## 1. Introduction

Lead (Pb) is a heavy metal found extensively distributed in the soil from which a great variety of products are made. Currently, the most common lead exposure sources in Mexico are the metallurgical industry emissions, battery recycling establishments, soil [1], and the use of glazed pottery for food preparation and storage, the latter source being the main form of exposure in Mexico [2].

The adverse effects of lead in humans have been widely studied [3–5]. The risk is greater to children, who absorb between 40 and 50% of the amount of lead consumed, while adults only absorb between 3 and 10% (ATSDR, 2012). On the other hand, fasting and nutritional deficiencies in calcium, iron, and zinc can increase gastrointestinal absorption of lead [6, 7]. Chronic lead poisoning can cause a decrease in IQ and mental development and attention disorders with blood lead levels (BLL) of <10 *μ*g/dL [8, 9]. Furthermore, in the late 2012, the Advisory Committee on Childhood Lead Poisoning Prevention of the CDC (ACCLPP) recommended decreasing the reference level from 10 to 5 *μ*g per dL explaining that there is enough scientific evidence that shows adverse effects on children with BLL below 10 *μ*g per dL [10].

Through the Federal Commission for Protection against Sanitary Risks (COFEPRIS), Mexico has implemented actions that study the existence of a lead-free glaze in the market, trained potters, and created a normative framework, ensuring that ceramic fired at low temperatures will not represent a health risk [11]. Important progress has been made over the years; nonetheless, there are still potters in Mexico that use lead oxide (also known as “Greta”) as glaze in low temperature glazed ceramic production, despite its poisonous effects. According to the COFEPRIS Potters Census, there are around 20 thousand people dedicated to this activity, although there is an important subregistry, because vast numbers of people conduct their activities for subsistence in marginalization and informality. On the other hand, the custom of using glazed cooking ceramic pots arises from deeply rooted Mexican traditions; the problem is that if pottery continues to be finished with lead glazes, leaching can occur when used for cooking or with acidic seasonings such as lemon or vinegar, combining Pb with the food that is going to be consumed by the user who can acquire chronic poisoning [12].

The few studies on lead poisoning in Mexican children associated with glazed ceramics have reported a prevalence of cases with lead levels above 10 *μ*g/dL [13, 14]. The persistence of lead poisoning in children arises out of the fact that some countries recognize and have identified and regulated sources of lead exposure but have not yet implemented monitoring and exposure prevention programs. Additionally, if the potential problem of poisoning has not been recognized, there are no surveillance programs nor researches on the matter. Consequently, public health authorities are unaware that there is still a risk of lead exposure or lead poisoning among children [15]. Therefore, this research seeks to identify the presence of lead in glazed ceramic utensils that are sold in a Mexican-US border city and are used for food preparation and cooking.

## 2. Methods 

A cross-sectional study on ceramic ware was conducted between November 2011 and November 2013. Pots were purchased from establishments that sold glazed ceramic pots in the Mexico-US border city of Tijuana, Mexico.

The stores were located in different geographic areas and different types of establishments were included (crafts markets, supermarkets, and business shops) with the purpose of having a diverse sample. Ceramics used to prepare or store food, such as pots, plates, and cups, were purchased. In order to decide how many containers should be purchased from each establishment, the owner was asked about the origin of the pieces. Each item was checked for design and manufacturer's inscriptions. Each pot purchased had a unique design and manufacturer.

A checklist was developed to explore the following characteristics of the purchased ceramic ware: presence of glaze, size, price, inscription warning of the presence of lead, place of purchase, date of purchase, and product origin.

To identify presence of lead in the pots, a lead verifier swab called LeadCheck Swabs was used. LeadCheck Swabs are recommended by the United States Environmental Protection Agency (EPA, 2013) and are used to detect lead on most surfaces, such as ferrous metal, wood, brick, cement, plastic, drywall, and plaster. This test can detect the presence of lead in concentrations as small as 1-2 *μ*g [16]. In the presence of lead, the swab color goes from pink to red [17, 18]. The Mexican Official Rule NOM-231-SSA1-2002 acceptable limit of lead solubility on flat pieces is 2 *μ*g/mL, on small hollow pieces 2 *μ*g/mL, on large hollow pieces 1 *μ*g/mL, and for utensils used to process food, beverages, cups, and jars 0.50 *μ*g/mL [19].

The test was carried out as recommended by the manufacturer (two minutes maximum). The pottery pieces were previously cleaned, the swab was activated, and the tube was shaked and squeezed until a bit of the yellow reagent came out at the end of the swab tip which indicated that it was ready for the test. The swab was then firmly rubbed inside the cooking pot for 30 seconds; if lead was present, the swab color changed from pink to red, giving a “positive” test for lead. The darker the red, the higher the lead content. If the swab tip color remained unchanged, lead was not detected and the test was considered negative. To control the quality of the test (as recommended by the manufacturers), a verifier was applied. If the test was negative, a test confirmation card was used; this card had three points containing a small amount of lead to confirm the lead-check reagent liquid reactivity. At the beginning of each test, the swab activation was confirmed with a control card.

The data are presented using simple descriptive statistical method like frequencies and proportions. The Fisher Exact Test, recommended for small samples, was used to calculate the statistical difference in proportions of lead-positive pots by price and by presence of a “Lead Free” label. Odds ratios and 95% confidence intervals were calculated to assess the risk of pots testing positive for lead. The statistical package utilized was SPSS version 17 for Windows.

## 3. Results 

Forty-one ceramic pots were purchased in 22 different establishments. Pots were purchased from all establishments selling glazed pottery products and located in different geographic areas of the city of Tijuana. The establishments included 13 crafts markets, 5 supermarkets, and 4 business shops. Thirteen pots were purchased in the northern part of the city, 12 in the southern, 8 in the center, 5 in the eastern, and 3 in the western. Only 4 of the establishments had pots with a label stating: “This cooking pot is lead-free” or “Lead Free, Food Use Safe.” Throughout each store, the “Lead-Free” pieces were mixed with the rest of the pots with no label. The rest of the visited establishments had pots without the “Lead-Free” label. 73.2% (30 out of 41) of the pots were lead-positive and 26.8% (11 out of 41) were lead-negative. Among the positives, 41.5% (17/30) were pink and 31.7% (13/30) red. Only 9.8% (4 out of 41) showed the label “Lead-Free.” The prices of the cooking pots were between $0.50 and $15.50 USD; there was no statistically significant association between the presence of lead and the pot price. Some cooking pots were more expensive and contained lead and some less expensive did not contain lead. No statistically significant differences were found regarding the size of the piece or the place of purchase. About 58.5% (24) of the glazed ceramic pieces were from the State of Jalisco and 24.4% (10) of the pieces were of unknown origin. Also, 26.8% of the swabs turned yellow, 31.7% red, and 41.5% pink (Table 1).

Only 4 of the cooking pots had no glaze and they were all (100%) lead-negative.

Therefore, of the 37 glazed pots, 81.1% (30 out of 37) were lead-positive and 18.9% (7 out of 37) were lead-negative. The pots with the label “This cooking pot is lead-free” or “Lead Free, Food Use Safe” were among the lead-negative pots. For the 33 remaining containers with glaze that did not show a “Lead-Free” label, 90.9% (30 of 33) were lead-positive and only 9.1% (3 out of 33) were lead-negative. Our conclusion is that cooking glazed pottery without a “Lead-Free” label has 1.6 more chances of containing lead (95% confidence interval = 1.0 to 2.5; *P* = 0.003).

## 4. Discussion 

The majority of glaze pots that did not show the “Lead-Free” label contained lead. This is different from what is currently expected. We must emphasize that the use of glazed pottery for cooking and food storage has been a significant predictor of elevated blood lead levels in the Mexican population [20, 21].

Mexico published its first regulation to set acceptable lead limits in glazed ceramics in 1994 (SSA, 1994) and has made great efforts to decrease the risk of lead exposure. Although about 4,500 potters have been trained through the National Fund for the Promotion of Crafts (FONART), few of them stopped using lead oxide because there is little availability of lead-free glaze in their comunities [11]. According to COFEPRIS, until the year 2007, actions had been implemented in 13 federal entities of Mexico [11]; nonetheless, the finding of lead in the majority of purchased clay pots in this study shows that lead-free techniques are not being followed, at least in the states where the sampled containers came from. On the other hand, it also implies that, in only 3 of the establishments, some of the products had the required “Lead-Free” label, which should be required for all the shops. In addition, in most of the establishments, sellers recommended that, prior to their use, the client should submerge the cooking pot in water for a while; others said that the cooking pots should be washed with acid or a home treatment, the latter being an erroneous belief that this treatment would prevent hazardous substances from being released. Experimental trials have demonstrated that it is not possible to eliminate exposure to lead in glazed ceramic vessels using home methods [12].

It is important to mention that the reagent used did not assess the exact concentration of lead. Even though there are more sophisticated laboratory techniques to measure lead content, according to the EPA, the method used in this study was effective in qualitatively identifying the presence of lead in painted surfaces [22] and indicated a concentration of lead depending on the reagent color change from pink to red [17, 18]. It was also observed that this reagent could give a false negative result on surfaces with high lead presence [23].

Another factor to be considered was that our sample was not random; however, pieces were obtained from different geographic areas of the city. Pieces were purchased from the most popular craft shops for local customers and foreign visitors. Despite the fact that the sample was small, care was taken to obtain pieces that represented most of those on sale, and if those came from the same manufacturer, it was considered unnecessary to buy more pieces.

The use of glazed ceramics is part of the Mexican culture; it represents a cultural trait, and, above all, it is an important job source for many Mexicans [12]. To completely eliminate this source of lead exposure, besides a stronger commitment to enforce regulations, it will be necessary to obtain an active response from an organized society in order to educate and increase awareness about the risks of using glazed ceramics in this most important target population.

## Figures and Tables

**Table 1:** Characteristics of the pots purchased in the establishments in the Mexico-US border city of Tijuana, 2013.

Item number	Type of store	Lead-free label	Estate of origin	Container type	Glazed?	Test color	Test result
1	1	No	Unknown	Pot	No	Yellow	Negative
2	1	No	Unknown	Pot	Yes	Pink	Positive
3	1	No	Unknown	Pot	Yes	Pink	Positive
4	1	No	Unknown	Casserole	No	Yellow	Negative
5	1	No	Jalisco	Casserole	Yes	Red	Positive
6	1	No	Jalisco	Casserole	Yes	Red	Positive
7	1	No	Jalisco	Cup	No	Yellow	Negative
8	1	No	Jalisco	Cup	Yes	Pink	Positive
9	2	No	Jalisco	Casserole	Yes	Pink	Positive
10	2	No	Jalisco	Casserole	Yes	Yellow	Negative
11	1	No	Unknown	Cup	Yes	Red	Positive
12	1	No	Unknown	Casserole	Yes	Pink	Positive
13	1	No	Unknown	Pot	Yes	Pink	Positive
14	1	No	Jalisco	Flat plate	Yes	Pink	Positive
15	1	No	Jalisco	Casserole	Yes	Pink	Positive
16	3	No	Unknown	Casserole	Yes	Red	Positive
17	3	No	Unknown	Pot	Yes	Pink	Positive
18	3	No	Jalisco	Casserole	Yes	Pink	Positive
19	3	No	Jalisco	Casserole	Yes	Pink	Positive
20	2	No	Edo.México	Cup	Yes	Yellow	Negative
21	2	No	Unknown	Cup	Yes	Pink	Positive
22	2	No	Edo.México	Pot	No	Yellow	Negative
44	1	No	Jalisco	Pot	Yes	Red	Positive
45	1	No	Jalisco	Pot	Yes	Red	Positive
46	1	Yes	Jalisco	Pot	Yes	Yellow	Negative
47	1	Yes	Jalisco	Pot	Yes	Yellow	Negative
48	1	No	Jalisco	Flat plate	Yes	Red	Positive
49	2	No	Jalisco	Casserole	Yes	Red	Positive
50	2	No	Edo.México	Casserole	Yes	Red	Positive
51	1	Yes	Jalisco	Casserole	Yes	Yellow	Negative
31	3	No	Jalisco	Casserole	Yes	Pink	Positive
32	3	No	Jalisco	Bowl	Yes	Pink	Positive
33	3	No	Jalisco	Cup	Yes	Red	Positive
34	1	No	Jalisco	Bowl	Yes	Pink	Positive
35	1	No	Jalisco	Bowl	Yes	Yellow	Negative
36	1	No	Jalisco	Pot	Yes	Red	Positive
37	1	No	Oaxaca	Small pot	Yes	Pink	Positive
38	1	No	Oaxaca	Casserole	Yes	Pink	Positive
39	1	No	Puebla	Bowl	Yes	Red	Positive
40	1	No	Puebla	Pitcher	Yes	Red	Positive
41	1	Yes	Jalisco	Pot	Yes	Yellow	Negative

Type of store: 1: craft market, 2: supermarket, and business shop: 3.
